# Cockroach Allergen Bla g 7 Promotes TIM4 Expression in Dendritic Cells Leading to Th2 Polarization

**DOI:** 10.1155/2013/983149

**Published:** 2013-09-24

**Authors:** Lingxiao Xu, Miaojia Zhang, Wenjing Ma, Shanshan Jin, Weijuan Song, Shaoheng He

**Affiliations:** ^1^Department of Rheumatology, the First Affiliated Hospital of Nanjing Medical University, Nanjing, Jiangsu 210029, China; ^2^Clinical Research Centre, the First Affiliated Hospital of Nanjing Medical University, Nanjing, Jiangsu 210029, China

## Abstract

As one of the most common sources of indoor aeroallergens worldwide, cockroach is important in causing rhinitis and asthma while the mechanisms underlying remain obscure. Since T helper (Th) type 2 polarization plays an important role in the pathogenesis of allergic diseases, we investigated the effect of Bla g 7, a pan-allergen from *Blattella germanica* (*B. germanica*), on Th polarization which is controlled by monocyte-derived dendritic cells (DCs). Challenged by recombinant Bla g 7 (rBla g 7), immature DCs obtained from human exhibited upregulated levels of TIM4, CD80, and CD86 and increased IL-13 secretion. Cocultured with CD4+ T cells, challenged DCs increased the ratio of IL-4+ versus IFN-*γ*+ of CD4+ T cells, suggesting a balance shift from Th1 to Th2. Moreover, antibodies against TIM4, CD80, and CD86 reversed the enhancement of IL-4+/IFN-*γ*+ ratio and alleviated the IL-13 release induced by rBla g 7, indicating that the Th2 polarization provoked by rBla g 7 challenged DCs is via TIM4-, CD80-, and CD86-dependent mechanisms. In conclusion, the present findings implied a crucial role of Bla g 7 in the development of cockroach allergy and highlighted an involvement of DCs-induced Th2 polarization in cockroach allergy.

## 1. Introduction

Studies on asthmatic patients and animal models of allergy have demonstrated that Th2 cells produce cytokines IL-4, IL-5, and IL-13 and thus contribute to development of asthma characterized by airway inflammation, mucus hypersecretion, and airway hyperresponsiveness [1]. However, the mechanisms through which naïve T cells differentiate to Th2 cells in response to allergens remain unclear.

DCs are a group of cells which possess the ability in dictating naïve CD4+ T cells differentiation to either Th1 or Th2 cells depending on ambient microenvironment [1, 2]. It is still an enigma how antigen-specific Th2 cells get skewed polarization and how they maintain a dominant status in allergy. Recent studies have showed that DCs express T cell immunoglobulin mucin domain (TIM)4 that ligates TIM1 on Th2 cells to promote Th2 cells development [3]. The report has shedded new light on the mechanism of allergic diseases. However, the factors which contributes to TIM4 upregulation in DCs remain unknown.

TIM is a new family of cell surface proteins that are potentially involved in the regulation of effector T cell responses [4]. Accumulated data suggest that several TIM molecules play critical roles in the regulation of Th1 and Th2 immune responses. Among them, DC-derived TIM4 has been found to be able to drive CD4+ T cells into Th2 cells [4, 5]. It has also been observed that the expression of TIM4 on DCs could be up-regulated dramatically upon activation [6, 7]. However, little is known about the actions of allergens in TIM4 upregulation on DCs.

Cockroaches have been identified to induce allergy in different regions of the world [8–11]. The most common cockroach species, which are frequently found in homes, are *B. germanica* and *Periplaneta americana* (*P. americana*) [12, 13]. Over the past decade, seven allergens of *B. germanica* (Bla g 1, Bla g 2, Bla g 4, Bla g 5, Bla g 6, Bla g 7, and Bla g 8) have been cloned [14–17]. Among them, Bla g 7 is a tropomyosin and has a highly cross-reactive pan-allergen to serum IgE. Since the structure of Bla g 7 is very similar to Per a 7 (a tropomyosin from *P. americana*), which has been previously reported to be able to activate the secretion of Th2 cytokine, IL-4, and IL-13, from P815 mast cell line [18, 19], we anticipated that Bla g 7 may also stimulate Th2 cytokine release from other immune cells. As DCs are the most powerful antigen presenting cells, which could induce Th2 polarization and intestinal allergy [7], the initiation factors through which DCs trigger CD4+ T cell differentiation remain unknown. Therefore, in this study, we hypothesized that Bla g 7 might affect DCs by promoting TIM4 expression in DCs and evoke CD4+ T cells to differentiate into Th2 cells subsequently. The results obtained here confirmed our assumption.

## 2. Materials and Methods

### 2.1. Reagents

TRIzol Reagent was purchased from Invitrogen (Carlsbad, CA, USA). ExScript RT reagent kit and SYBR *Premix Ex Taq* (perfect real time) were obtained from TaKaRa (DaLian, China). PE-conjugated mouse antihuman IL-4, CD80, CD86, and FITC-conjugated mouse antihuman IFN-*γ* antibodies were obtained from eBioScience (Los Angeles, CA, USA). Antihuman I-*κ*B, phospho-I-*κ*B, NF-*κ*B, and phospho-NF-*κ*B antibodies were purchased from Cell Signaling Technology Inc. (Beverly, MA, USA). Anti-TIM4 antibody was purchased from R&D Systems (San Diego, USA). GM-CSF, and IL-4 was purchased from Peprotech (Rocky Hill, USA). rBla g 7 and its monoclonal antibody were prepared in our laboratory as shown previously [20]. Most of other reagents such as salt and buffer components were of analytical grade and obtained from Sigma-Aldrich (St. Louis, MO, USA).

### 2.2. Culture and Challenge of DCs

Peripheral blood mononuclear cells (PBMCs) from 6 healthy volunteers were isolated by Ficoll centrifugation. CD14+ monocytes were then purified by CD14+ magnetic microbeads (Miltenyi Biotec, Germany) according to the manufacturer's instruction. They were cultured in RPMI-1640 supplemented with 10% fetal bovine serum, antibiotics, GM-CSF (10 ng/mL), and IL-4 (5 ng/mL). The further addition of the cytokines was carried out every other day. After 5 days of culture, immature DCs were harvested for use. 

For challenge experiments, cells were exposed to various concentrations (10–1000 ng/mL) of rBla g 7 with or without anti-TIM4 antibody (10 ng/mL), or 100 ng/mL of LPS (as positive control). At 48 h following incubation, the culture plates were centrifuged at 450 ×g for 10 min at 25°C. After the supernatants being collected and stored at −80°C, the cell pellet containing approximately 5 × 10^6^ matured DCs was collected for Western-blot, flow cytometry, and real-time PCR analyses. Some DCs were pulsed with 100 ng/mL LPS and 1 *μ*g/mL rBla g 7 for 24 h, washed, and then cultured with CD4+ T cells for 2 days.

In order to investigate the influence of CD80 and CD86 molecules on rBla g 7-activated DCs induced polarization of CD4+ T cells, rBla g 7-activated DCs were preincubated with CD80 and CD86 blocking antibodies respectively, for 30 min at 37°C before being cocultured with CD4+ T cells.

### 2.3. Isolation of CD4+ T Cells and Coculture with DCs

CD4+ T cells were purified from the PBMC (donated by 6 healthy volunteers) with CD4+ magnetic microbeads according to the manufacturer's instruction. Isolated CD4+ T cells were then mixed with rBla g 7-activated DCs at a ratio of 10 : 1 with or without TIM4 blocking antibody (10 ng/mL). The cocultured two types of cells were maintained in RPMI-1640 culture medium supplemented with 10% FBS and antibiotics for 48 h at 37°C. Following centrifugation, the supernatant was collected, and levels of IFN-*γ* and IL-13 in the supernatant were measured by ELISA. The cell pellet was resuspended, and intracellular expression levels of IFN-*γ* and IL-4 were measured by flow cytometry analysis.

### 2.4. Western Blot Analysis

After being challenged with 10, 100 and 1000 ng/mL rBla g 7 or medium alone for 48 h and challenged with 1000 ng/mL rBla g 7 (or medium alone) for 20 min, 40 min, and 60 min, the purified DCs were lysed as previously described [19]. Proteins were separated by 12% SDS-PAGE and transferred onto a nitrocellulose membrane. Immunoreactive proteins were detected by incubating blots with specific Abs. Densitometry analysis of immunoblots was carried out by using Quantity One software (Bio-Rad, USA). The relative level of phosphor-I-*κ*B and NF-*κ*B was expressed as the ratio to I-*κ*B and NF-*κ*B, and TIM4 was expressed as the ratio to GAPDH, an internal control.

### 2.5. Quantitative Real-Time PCR

Total RNA from the DCs was isolated using TRIzol reagent. The cDNA was prepared by ExScript RT reagent kit according to the manufacturer's instruction and then was amplified by PCR with specific primers. Briefly, real-time PCR was performed by using SYBR *Premix Ex Taq* on a Sequence Detection System (Eppendorf, Germany). The thermal cycling conditions included an initial denaturation step at 95°C for 30 s, followed by 40 cycles of 5 s at 95°C and 30 s at 60°C. Primer sequences for human TIM4, IL-12, and IL-13 and GAPDH are summarized in Table 1. Relative gene expression was determined by the 2^−ΔΔct^ method. 

### 2.6. Flow Cytometry Analysis

DCs were pelleted by centrifugation at 450 ×g for 5 min and then fixed and permeabilized by using a cell fixation/permeabilization kit (BD Pharmingen). Briefly, thoroughly resuspended cells were added in 100 *μ*L of BD Cytofix/Cytoperm solution and incubated for 30 min at 4°C. DCs were then incubated with PE-conjugated CD80, and CD86 and CD4+ T cells were incubated with FITC-conjugated IFN-*γ* and PE-conjugated IL-4 monoclonal antibody or isotope control, respectively (at a final concentration 4 *μ*g/mL), at 4°C for 30 min. After washing, cells were analyzed on a fluorescence-activated cell sorting (FACS) Arial flow cytometer with FlowJo software (TreeStar, San Carlos, CA, USA).

### 2.7. ELISA

Levels of IL-13 and IL-12p70 (Excell China) in the culture supernatant of DCs or IL-13 and IFN-*γ* in the culture supernatant of T cells were measured by ELISA kits according to manufacturer's instructions.

### 2.8. Statistical Analysis

Data are expressed as mean ± SEM for the indicated number of independently performed duplicated experiments. Statistical significance between means was analyzed by the Student's *t*-test utilizing the SPSS 13.0 version. *P* < 0.05 was taken as statistically significant.

## 3. Results

### 3.1. Upregulation of TIM4 Expression on DCs by rBla g 7

**Figure 1:**
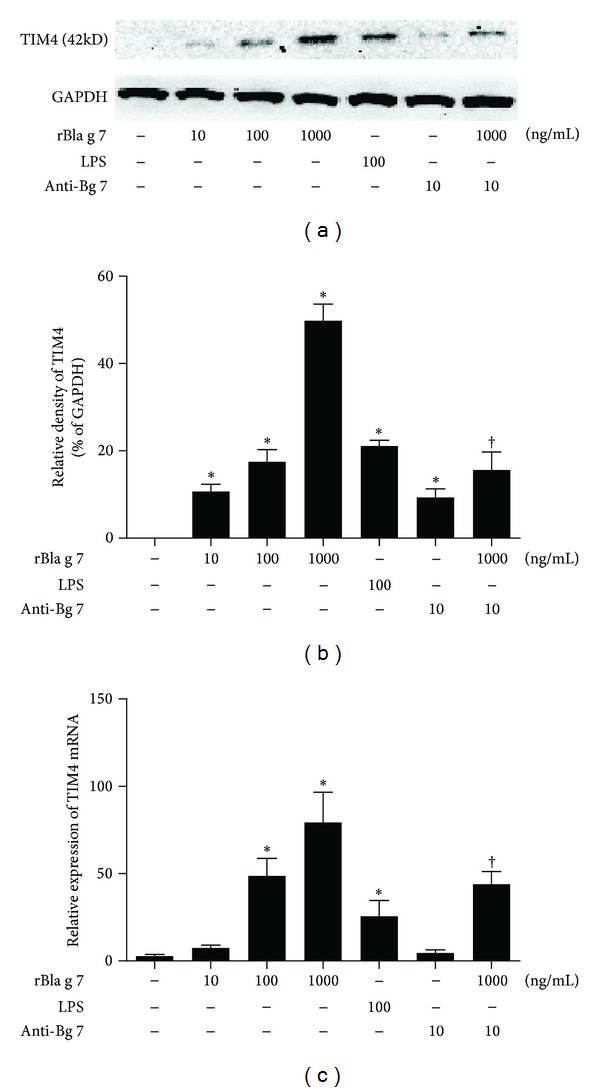
rBla g 7 induced the expression of TIM4 in dendritic cells (DCs). Various concentrations of rBla g 7 or LPS (100 ng/mL) were incubated with DCs for 48 h at 37°C. (a) Western blot analysis of TIM4 expression in DCs, (b) density analysis of TIM4 expression in Western blot, and (c) real-time PCR to determine the relative expression of TIM4 mRNA in DCs. The expression values were normalized against GAPDH mRNA, and relative gene expression was determined by the 2^−ΔΔct^ method. The data were represented as the mean ± SEM for four separate experiments. *P < 0.05 in comparison with medium alone control. ^†^ *P* < 0.05 compared with the response to the corresponding uninhibited control.

Previous reports indicated that DCs express TIM4 that plays a critical role in inducing peripheral Th2 polarization [4, 21]. However, the factors in upregulating TIM4 expression in DCs remain unknown. As shown in Western blot, the expression of TIM4 in DCs was significantly increased in response to rBla g 7 in a dose-dependent manner (Figures 1(a) and 1(b)). Similarly, TIM4 mRNA expression in human DCs was enhanced by up to approximately 28.2-fold assessed by realtime PCR analysis when the cells were cultured with various concentrations of rBla g 7 (Figure 1(c)). Once being added at the same time with rBla g 7, anti-Bla g 7 antibody was able to diminish rBla g 7 induced TIM4 protein and mRNA expression though it itself induced modest TIM4 expression in DCs (Figure 1).

### 3.2. Induction of Maturation and Activation of DCs by rBla g 7

**Figure 2:**
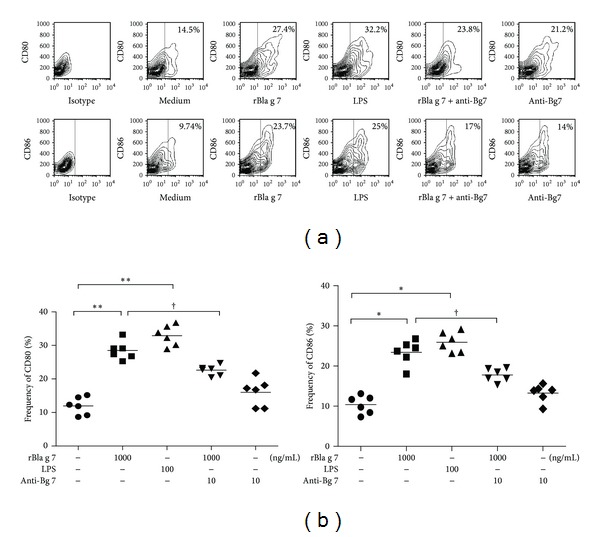
rBla g 7 induced expression of CD80 and CD86 in dendritic cells (DCs)s. DCs were incubated with rBla g 7 (1000 ng/mL) or LPS (100 ng/mL) for 48 h at 37°C before being analysed by flow cytometry. (a) Numbers within the large gated regions indicate the percentage of matured cells among total cells. (b) The mean ± SEM data represented the percentage of CD80+ or CD86+ DCs for four separate experiments. *P < 0.05 in comparison with medium alone control. ^†^ *P* < 0.05 compared with the response to the corresponding uninhibited control.

Expression of high levels of CD80 and CD86 represents the markers of mature DCs [22]. CD80 and CD86 mediate the necessary costimulatory signals to T cells, which results in Th1/Th2 cell polarization. It has also been reported that enhanced expression of CD80 and CD86 was observed on human monocyte-derived dendritic cell's (MoDCs) during maturation [23], and we therefore investigated expression of CD80 and CD86 on DCs in the present study. rBla g 7 induced approximately 2.4- and-2.2 fold upregulated expression of CD80 and CD86 on immature DCs, indicating the maturation of these immature DCs. The phenotypic changes induced by rBla g 7 were comparable to those elicited by LPS (100 ng/mL), suggesting that rBla g 7 is a potent stimulus of DC maturation and activation. Once being added at the same time with rBla g 7, anti-Bla g 7 antibody was able to diminish rBla g 7 induced CD80 and CD86 expression (Figure 2). 

### 3.3. Induction of Enhanced Expression of IL-13 by rBla g 7

**Figure 3:**
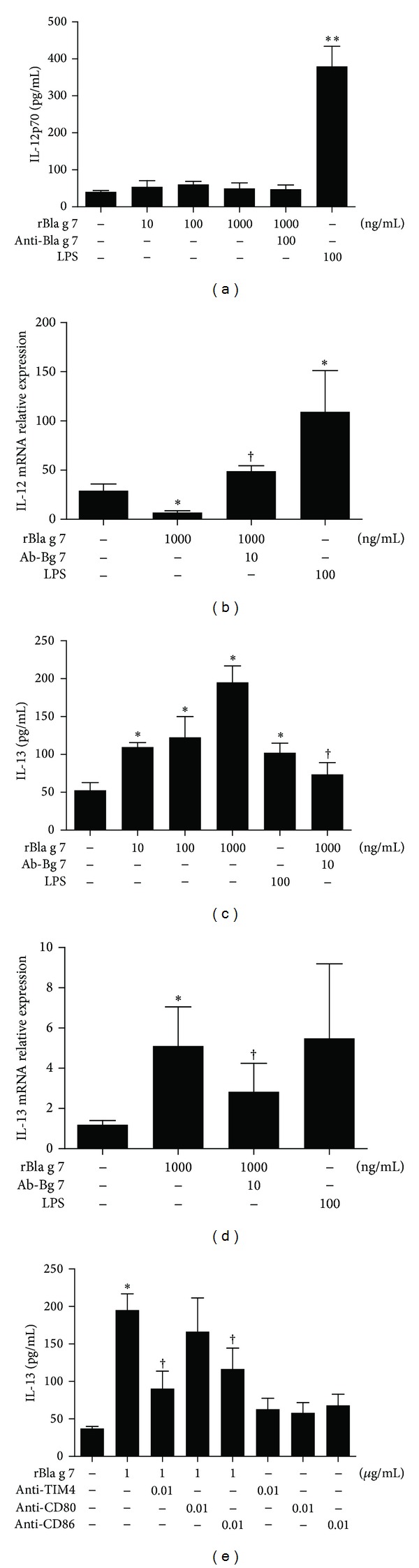
rBla g 7 induced release of IL-13 from dendritic cells (DCs). DCs were incubated with various concentrations of rBla g 7 (ng/mL) with or without its specific blocking antibody (Ab-Bg 7, 10 ng/mL), or with LPS for 48 h at 37°C. The levels of IL-12 (a) and IL-13 (c) in the culture supernatant were determined by ELISA, and the expression of IL-12 (b) and IL-13 (d) mRNAs was assessed by real-time PCR. The influence of TIM4, CD80, and CD86 blocking antibodies on rBla g 7 provoked IL-13 release was also examined (e). The data were represented as the mean ± SEM for four separate experiments. *P < 0.05 in comparison with medium alone control. ^†^ *P* < 0.05 compared with the response to the corresponding uninhibited control.

IL-13 is a well-known Th2 cytokine, which contribute's greatly to the development of allergic inflammation. It has been shown that IL-13 is not only secreted from Th2 cells, but also released from basophils [24] and epithelial cells [25]. However, little is known about the influence of allergen on IL-13 secretion from DCs. In the present study, rBla g 7 induced a dose-dependent secretion of IL-13 from DCs, which was partially inhibited by TIM4 blocking antibody (Figure 3(c)), and CD80 and CD86 blocking antibodies (Figure 3(e)). rBla g 7 also up-regulated the expression of IL-13 mRNA in DCs (Figure 3(d)). In the parallel experiments, rBla g 7 at the doses tested failed to show any effect on IL-12 protein (Figure 3(a)) or mRNA expression (Figure 3(b)) in DCs.

### 3.4. rBla g 7 Activates NF-*κ*B Signaling Pathway in DCs

**Figure 4:**
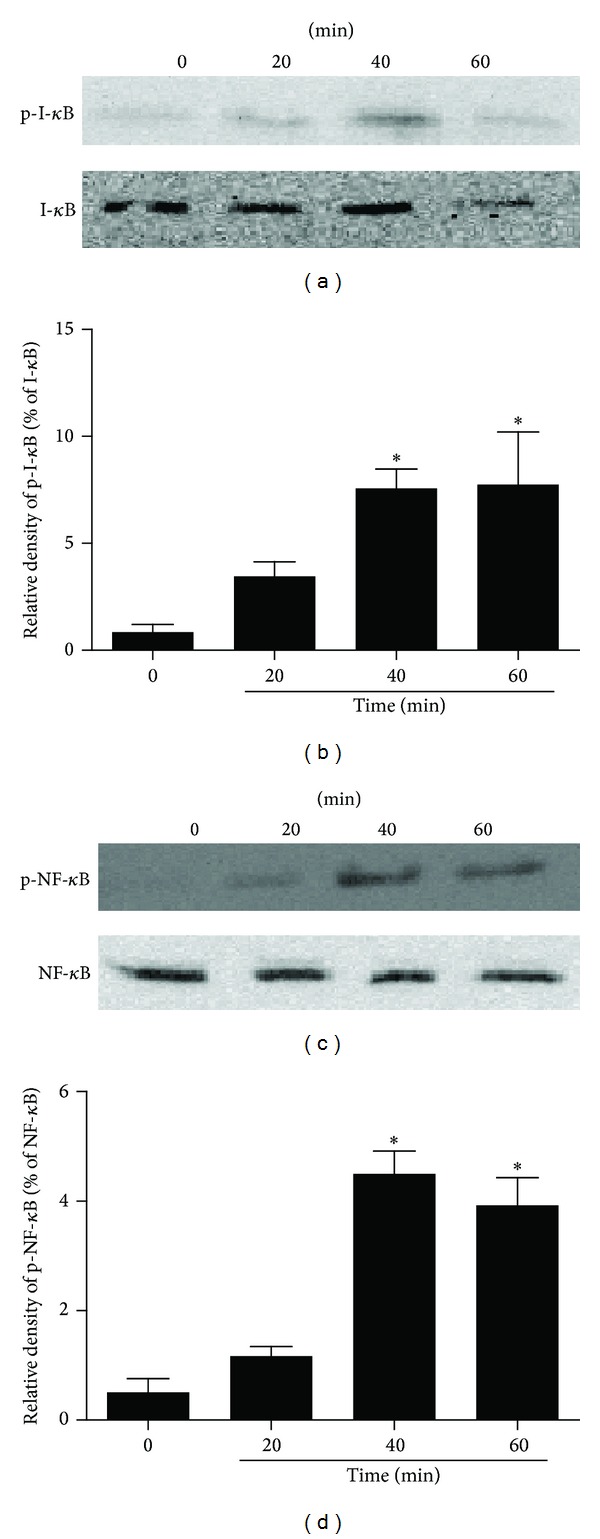
Time courses of recombinant Bla g 7 (rBla g 7) induced I-*κ*B and NF-*κ*B activation in dendritic cells (DCs). (a, c) rBla g 7 at 1 *μ*g/mL was incubated with DCs at 37°C for 0, 20, 40, and 60 min, respectively. Protein levels were detected by immunoblot analysis with specific antibodies. Western blots at the top of each panel are from a typical experiment. (b, d) Bar graphs are the quantified results expressed as mean ± SEM of I-*κ*B, p-I-*κ*B, NF-*κ*B, and p-NF-*κ*B levels from three independent experiments. *P < 0.05 versus 0 min.

NF-*κ*B acts as a mater switch for allergic inflammation disease [26]. Therefore, we examined I-*κ*B and NF-*κ*B activation in DCs in the presence of rBla g 7. The results revealed that rBla g 7 triggered phosphorylation of I-*κ*B and NF-*κ*B in DCs initiated at 20 min following incubation and lasted for 40 and 60 min, respectively (Figure 4).

### 3.5. Induction of Th2 Polarization by rBla g 7-Activated DCs

**Figure 5:**
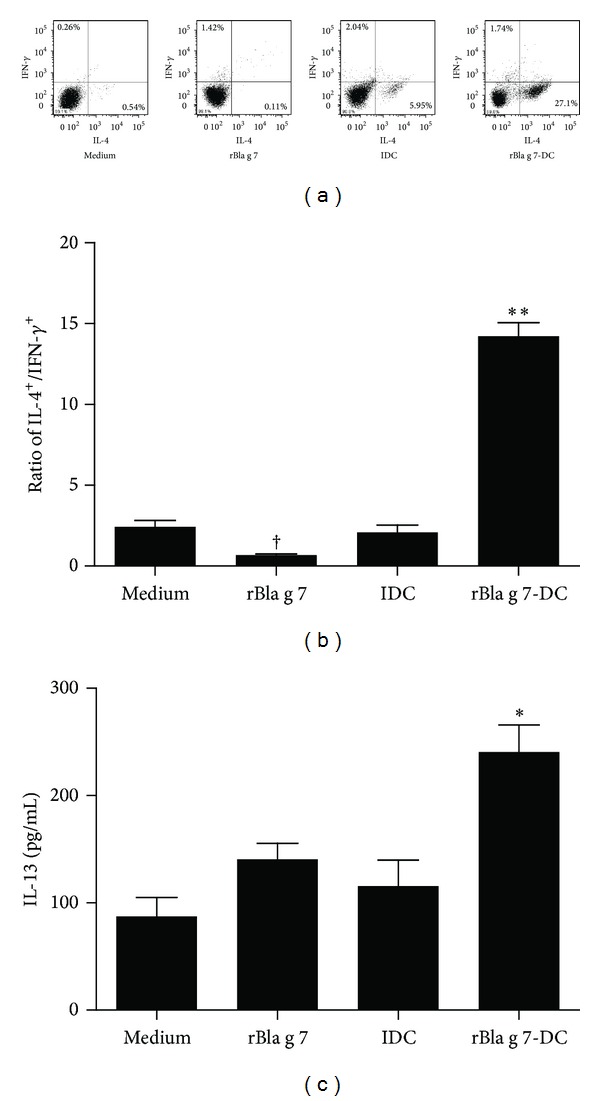
Induction of Th2 polarization by rBla g 7-stimulated dendritic cell (DC)s. Isolated CD4+ T cells were cocultured with immature DCs (iDC), rBla g 7-activated DCs (rBla g 7-DCs), or rBla g 7-pulsed alone (rBla g 7) for 48 h at 37°C before being harvested for analysis. (a) Flow cytometry analysis of intracellular expression of IFN-*γ* and IL-4 by using the fluorescent-labeled antibodies. It was noticed that the number of IL-4+ cells in the lower right quarters of IDC and rBla g 7-DCs groups was increased. (b) The ratio of IL-4+ CD4+ T cells versus IFN-*γ*+ CD4+ T cells. (c) Levels of IL-13 in the culture supernatants determined by ELISA. The data were represented as the mean ± SEM for four separate experiments. *P < 0.05, **P < 0.01 in comparison with medium alone control. ^†^ *P* < 0.05 for decreased response in comparison with control.

Induction of Th2 polarization is one of the crucial steps of sensitization process in allergy. However, the mechanism of cockroach allergen induced Th2 polarization remains uninvestigated. We found that coculture of rBla g 7-activated DCs with isolated CD4+ T cells induced Th2 polarization of helper T-cells as shown by enhanced ratio of IL-4+ cells versus IFN-*γ*+ cells (14.23 ± 0.82), which was approximately 7-fold higher than the ratio for immature DCs (2.082 ± 0.46) (Figures 5(a) and 5(b)). Moreover, coculture of rBla g 7-activated DCs with isolated CD4+ T cells resulted in the elevated level of IL-13 in the culture supernatant (Figure 5(c)).

### 3.6. Inhibition of rBla g 7-Activated DCs Induced Th2 Polarization by TIM4, CD80, and CD86 Antibodies

**Figure 6:**
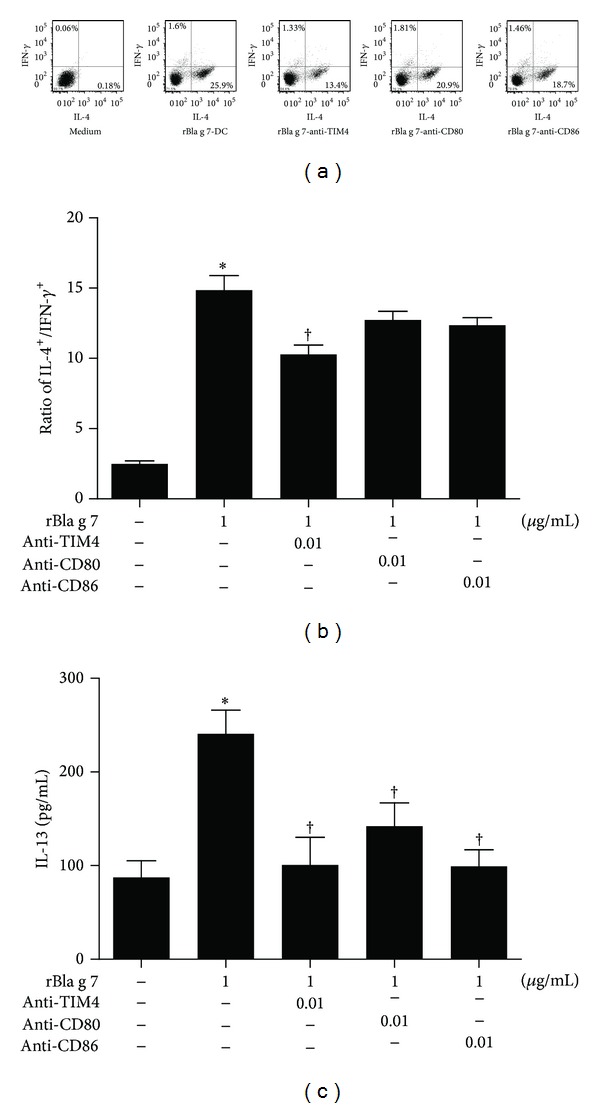
Inhibition of rBla g 7-stimulated dendritic cell (DC)s induced Th2 polarization by antibodies against TIM4, CD80, and CD86. In (a), isolated CD4+ T cells were cocultured with DCs treated with rBla g 7 (1 *μ*g/mL) alone (rBla g 7-DCs) or treated with rBla g 7 and anti-TIM4 (10 ng/mL, rBla g 7-anti-TIM4), anti-CD80 (rBla g 7-anti-CD80) or anti-CD86 antibodies (rBla g 7-anti-CD86) before flow cytometry analysis of intracellular expression of IFN-*γ* and IL-4 being performed. (b) The ratio of IL-4+ CD4+ T cells versus IFN-*γ*+ CD4+ T cells. (c) Levels of IL-13 in the supernatants of DCs determined by ELISA. The data were represented as the mean ± SEM for four separate experiments. *P < 0.05 in comparison with medium alone control. ^†^ *P* < 0.05 compared with the response to the corresponding uninhibited control.

Since DC-derived TIM4 has been reported to drive CD4+ T cells into Th2 cells [4, 5], it may mediate rBla g 7-induced Th2 polarization. Indeed, TIM4 antibody reduced rBla g 7-activated DCs induced IL-4 expression of CD4+ T cells by approximately 48% (Figure 6(a)), significantly diminished the ratio of IL-4+ versus IFN-*γ*+ cells (Figure 6(b)), and decreased the IL-13 level in the culture supernatant of cocultured rBla g 7-activated DCs and isolated CD4+ T cells (Figure 6(c)). In addition, CD80 and CD86 antibodies also inhibited rBla g 7-induced Th2 polarization when they were added to rBla g 7-activated DCs for 30 min before co-culturing with isolated CD4+ T cells (Figure 6).

## 4. Discussion

Although Bla g 1 [27] and Bla g 2 [28] were employed as markers of *B. germanica*, little is known about the profile of Bla g 7 in proinflammatory actions. In the present study, we found for the first time that Bla g 7 could potently induce TIM4 expression on DCs. Since activated DCs highly express TIM4 [7] which plays a critical role in T cell proliferation [4] and Th2 cell development [29], and DCs are mainly localized at the interface between epithelium and environment, and we believe that inhalant allergen Bla g 7 might initiate the sensitization process of allergy by activating DCs [30, 31].

On the other hand, Bla g 7 could also induce DCs maturation assessed by the levels of CD80 and CD86. As CD80 and CD86 have been found to mediate the necessary costimulatory signals to evoke Th2 polarization [32] involved in allergy, CD80 and CD86 upregulation induced by rBla g 7 in DCs may contribute to the subsequent allergic responses. A recent study demonstrated that a *P. americana* allergen Per a 10 induced significant CD86 upregulation on DCs, and provoked significantly low IL-12 but high IL-4 and IL-5 release from DCs [32]. Another report also confirmed that CD11c+ MHCII+ DCs within mediastinal lymph nodes expressed maturation markers such as CD80 and CD86, which supports our observation [33]. 

However, unlike Per a 10 and fungal allergens [34], rBla g 7 was found to only downregulate the level of IL-12 mRNA, but not IL-12 secretion from DCs. Instead, it induces IL-13 mRNA and protein upregulation from DCs. Since IL-13 serves as a foremost cytokine [35] and provides a first and crucial signal for class switching of B cells to produce IgE [36], our finding implicates that rBla g 7 may induce B cell to produce IgE [37] without activating Th2 cells. The previous studies have demonstrated that grass pollen and birch pollen were able to induce IL-13 production from human DCs [38], and that diesel-enriched particulate enhanced IL-13 secretion from human DCs [39] may support our view above. Moreover, our findings that antibodies against TIM4 and CD86, but not CD80 blocked rBla g 7-induced IL-13 production in DCs, indicated that the effects of rBla g 7 are via a TIM4 and CD86 dependent mechanism. Although high levels of CD80 and CD86 represent the DCs maturity [22], expression of CD80 and CD86 under the same stimulation respond differently as shown in the present study. Similar observation was reported previously that reduced expression of CD80 and CD86 was observed in the majority of DCs in vitro while a significantly increased CD86 expression was found in a subpopulation of DCs exposed to Maxadilan [40]. NF-*κ*B is a mater switch for allergic inflammation disease [26]. Induction of phosphorylation of I-*κ*B followed by NF-*κ*B activation by rBla g 7 indicates that NF-*κ*B translocation into nucleus may also be involved in maturation of DCs and IL-13 release from DCs. The previous report that NF-*κ*B1 expression within DCs is required to promote optimal Th2 responses following exposure to eggs [41] may support our observation above.

Although DCs from allergic individuals preferentially induce a Th2-type response, the mechanism underlying by which naïve CD4+ T cells develop to Th2 cells and how this process is out of control is a critical point to elucidate the mechanism of allergy. Using the current experiment model, we demonstrated for the first time that cockroach allergen Bla g 7 preferably induces naïve CD4+ T cells to become Th2 cells via DCs. Since antibodies against TIM4, CD80, and CD86 blocked rBla g 7-activated-DCs induced IL-13 release from CD4+ T cells, the actions of rBla g 7 on IL-13 secretion from CD4+ T cells are likely via TIM4, CD80, and CD86 dependent signaling pathways in DCs. However, the antibodies against TIM4, but not CD80 and CD86, blocked rBla g 7-activated-DCs induced IL-4 upregulation in CD4+ T cells indicates that the actions of rBla g 7 on IL-4 upregulation are likely via a TIM4, but not CD80 and CD86 pathway in DCs. Obviously, the involvement of different signaling pathways of DCs in the induction of IL-13 and IL-4 upregulation is still required to be further studied. 

In conclusion, we demonstrated in this study that cockroach allergen rBla g 7 induces DC-dictated Th2 polarization of CD4+ T cells through TIM4, CD80, and CD86 dependent mechanisms. Since Th2 cells are pivotal factors in allergy, Bla g 7 seems likely to play an important role in the development of cockroach allergy.

## Figures and Tables

**Table 1:** Primer sequences used in quantitative real-time PCR.

Primer	Sequence	Size of product (bp)
TIM4 Sense	5′-ACAGGACAGATGGATGGAATACCC-3′	169 bp
Antisense	5′-AGCCTTGTGTGTTTCTGCG-3′	
IL-12 sense	5′-AGTTCAGCCTCAGAATGCAA-3′	208 bp
Antisense	5′-TAACAGCCATGTGAGAAGCA-3′	
IL-13 sense	5′-TGAGGAGCTGGTCAACATCA-3′	75 bp
Antisense	5′-CAGGTTGATGCTCCATACCAT-3′	
GAPDH, Sense	5′-AGAAGGCTGGGGCTCATTTG-3′	257 bp
Antisense	5′-AGGGGCCATCCACAGTCTTC-3′	
